# Single‐ and Three‐Photon Ionization of $N_{2}$ in Presence of Fano Resonances

**DOI:** 10.1002/jcc.70067

**Published:** 2025-03-11

**Authors:** Vinay Pramod Majety, Armin Scrinzi

**Affiliations:** ^1^ Department of Physics and CAMOST Indian Institute of Technology Tirupati Yerpedu India; ^2^ Department of Physics Ludwig Maximilian University Munich Germany

**Keywords:** computational methods, nitrogen molecule, photoabsorption, time‐dependent Schrodinger equation

## Abstract

We report single‐ and three‐photon ionization cross‐sections of the $N_{2}$ molecule in the region of the Hopfield series of doubly excited states. Results are obtained by solving the time‐dependent Schrödinger equation in a hybrid basis combining neutral and ionic CI states with a fully numerical basis. Contributions to the spectrum during and after the interaction are obtained using the tSurff and iSurf methods. Calculations at arbitrary molecular orientation and details of the spectral calculation are presented. For single‐photon ionization synchrotron data is reproduced. For three‐photon ionization we find a pronounced change of resonance line shape when going from single‐ to three‐photon transitions.

## Graphical abstract

With upcoming high‐intensity attosecond light sources new theoretical approaches for multi‐photon interactions with molecular systems in the non‐perturbative and multi‐photon regime are needed. Solutions of the time‐dependent Schrödinger equation provide all this, but the massive involvement of continuum states necessitates a combination of Gaussian with purely numerical bases. A range of new methods and their implementation in the open source code package tRecX is demonstrated for multi‐photon absorption at the Hopfield series of N2 at arbitrary polarization direction. Good agreement with experiment is found and pronounced dependence of line‐shape on multi‐photon order is observed.

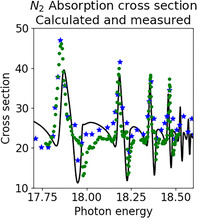

## 1 Introduction

Photoionization that involves both direct bound‐continuum transitions and an indirect path through an intermediate doubly excited state creates resonance peaks with characteristic, asymmetric line shapes that were first explained by Fano [1]. In the original model of Fano, the functional form of the line shape is universal for any perturbative process leading to ionization along two such paths, and it is usually studied in single‐photon ionization processes. In recent years, Fano resonances have attracted renewed interest in the context of attosecond physics, where ionization by an attosecond pulse can be modified by a simultaneous additional field. In Reference [2] that was studied with the Helium atom, where the line shape of single‐photon ionization by a pulse in the extreme ultraviolet (XUV) was modified by the presence of a laser pulse at the near‐infrared (NIR) wavelength of 800 nm, resulting in the change from Fano to Lorentzian line shape. More generally, the attosecond dynamics in the vicinity of Fano and shape resonances [3, 4] has been investigated.

Accurate data on photo‐absorption near the Hopfield series of doubly excited Rydberg states of $N_{2}$ has been thoroughly investigated by synchrotron absorption spectroscopy, (e.g., References [5, 6]). With ever increasing intensities of short wave‐length light sources, currently up to $≲10^{13}\;{\text{W/cm}}^{2}$ [7, 8], going beyond the basic single‐photon process to multi‐photon ionization is coming into reach for experiments. This motivated a recent study [9] in atoms, where we found, similar to the XUV+NIR setting, the line shape to change to the Lorentzian with increasing multi‐photon order.

For $N_{2}$, there exist early calculations on photoabsorption cross‐sections obtained in random phase approximation [10] and Møller–Plesset perturbation theory [11]. Resonances were computed recently using R‐matrix theory [12], XCHEM [13] and complex variable coupled cluster theory [14]. Compared to atoms, in molecules the orientation of the molecular axis relative to laser polarization enters as an additional parameter and a more complex excitation structure appears. For theory, the loss of rotational symmetry becomes a major technical challenge. A first thorough study of perturbative photo‐ionization of $N_{2}$ was presented in [13].

Multi‐photon ionization can no longer be described in first‐order perturbation theory using only matrix elements of the interaction between the initial ground and final resonant and continuum states. Rather, for n‐photon ionization it involves one or more intermediate levels of “virtual” states, or, putting it differently, multiple applications of the Green's function of the field‐free Hamiltonian $H_{0}$ $\lim_{\epsilon ↓0}{\left[{\hat{H}}_{0}-(E-m\hbar \omega )-i\epsilon \right]}^{-1}$ for all m<n at laser frequency ω, where E is the final state energy, see, for example, [15, 16]. Alternatively, one can solve the time‐dependent Schrödinger equation for a single, spectrally broad pulse and analyze the resulting wave packet. We used this approach to study multi‐photon Fano line shapes in Helium and Neon atoms, Reference [9]. Solutions of the time‐dependent Schrödinger equation were obtained by the tRecX‐haCC package developed in our group [17, 18]. The package employs the tSurff [19, 20] method for calculating photo‐emission. Due to technical limitations, a demonstration of how to compute $N_{2}$ spectra with tRecX‐haCC [18] was limited to single‐photon processes and parallel alignment of the laser polarization with the molecular axis.

In the present work we proceed from single‐ to multi‐photon ionization of $N_{2}$ and to arbitrary alignment of the molecular axis. In the single photon case, we reproduce results found in literature. In the three‐photon case, we find a dramatic change in line shapes compared to the single‐photon transition, just as for atoms [9]. The effect is most pronounced in parallel alignment, and less so with perpendicular alignment.

Below, before presenting the results, we summarize the methods and include details of the construction of operators in the hybrid CI and numerical bases, and the technical advancements of tRecX‐haCC that enabled the present calculations. After a section with literature comparisons for single‐photon absorption, we conclude with the new features seen in the multi‐photon case.

## 2 Methods and Implementation

### 2.1 The Hacc Expansion

In the hybrid antisymmetrized Coupled Channels (haCC) method one solves the time‐dependent Schrödinger equation (atomic units (au) $\hbar =m_{e}=e^{2}=1$ used throughout, unless indicated otherwise)

(1) $$i\frac{d}{dt}|\Psi (t)\rangle =\left[H_{0}+H_{I}(t)\right]|\Psi (t)\rangle$$

for an N‐electron Hamiltonian $H_{0}$ of a molecule with fixed nuclear positions and laser‐electron interaction $H_{I}(t)$ by an ansatz for |Ψ(t)⟩ in the form

(2) $$\left|\Psi (t\right)\rangle =\sum_{\mathrm{34𝒩}}|\mathrm{34𝒩}\rangle c_{\mathrm{34𝒩𝒩}}(t)+\sum_{A}\left\{\sum_{i=0}^{I-1}a_{i}^{†}|A\rangle c_{i}^{A}(t)+\sum_{\alpha }a_{\alpha }^{†}|A\rangle c_{\alpha }^{A}(t)\right\}$$

One employs neutral |3434𝒩⟩ and ionic |A⟩ CI bound state functions, which both are constructed from the same set of molecular orbitals $a_{i}^{†}|\rangle =|i\rangle ,i=0,\text{…}\;,I-1$ with their fermionic creation operators $a_{i}^{†}$. This is augmented by fully numerical basis functions $a_{\alpha }^{†}|\rangle =:|\alpha_{⊥}\rangle$. All time‐dependence is in the complex expansion coefficients $c_{\mathrm{3434𝒩}}(t),c_{i}^{A}(t)$, and $c_{\alpha }^{A}(t)$. In the present calculation we only use the ground state for 34𝒩. Adding more neutral CI states can be helpful, when these are strongly correlated and therefore poorly represented by the channel functions. The summation over α refers to the indices of a spherical expansion, which is specified below.

The rational of this expansion is that the correlated ground states and possibly some excited neutral states |3434𝒩⟩ play a key role. During ionization, ionic ground and excited states |A⟩ participate in the dynamics and after ionization they define the ionization channels. One electron is allowed to break away from the CI bound state setting and can cover an essentially unrestrained range of continuum states. The unrestricted representation of the single‐electron continuum provides a correct description of outgoing scattering on a coupled channels level. It can also describe a wide range of final photoelectron states. This is of minor importance in a perturbative situation, but it is essential in strong‐field, genuinely non‐perturbative processes, where emission spectra are inherently broad. Similarly, in strong laser fields, the continuum electron contributes to transient polarization that impacts on the ionization thresholds and crucially changes ionization rates.

A numerical basis |α⟩ is constructed as a standard spherical expansion

(3) $$\left|\alpha \rangle :=|m_{\alpha },l_{\alpha },n_{\alpha },j_{\alpha }\rangle =|Y_{m_{\alpha }l_{\alpha }}\rangle |\xi_{j_{\alpha }}^{n_{\alpha }}\right\rangle$$

where $\left\langle \phi ,\theta |Y_{ml}\rangle =Y_{ml}(\phi ,\theta \right)$ are spherical harmonics and $\left\langle r|\xi_{j_{\alpha }}^{n_{\alpha }}\right\rangle$ are FE‐DVR radial functions [21, 22]. For given n, the radial functions $\left\{\xi_{j}^{n},j=1,\text{…}\;,J_{n}\right\}$ have their support on an interval $\left[r_{n},r_{n+1}\right]$, where the $r_{n}$ can be freely chosen, except for $r_{0}=0$. For the last boundary one can also choose r=∞ (see Section 2.4.1). The size of $J_{n}$ and of the $Y_{ml}$‐angular expansion can be different for each n. More details are given in Reference [18].

Formally the spherical expansion (3) is complete, but it is inefficient in the vicinity of off‐center nuclei. The addition of all molecular orbitals |i⟩ to the single‐electron basis greatly improves the expansion near nuclei. The $\left|\alpha_{⊥}\right\rangle$ used in Equation (2) are orthogonalized to all |i⟩ as

(4) $$\left|\alpha_{⊥}\rangle =\left(1-\overset{I-1}{\underset{i=0}{\sum }}|i\rangle \langle i|\right)|\alpha \right\rangle$$

We control convergence of off‐center scattering without exceedingly bloating the α‐basis by using high FE‐DVR grid density at radial intervals that contain nuclei and by enhancing angular expansion on those intervals. This is done through standard input options of the tRecX code [18].

Apart from removing the formal linear dependence, the orthogonalization has the benefit that the $a_{\alpha }^{†}|A\rangle$ basis is strictly orthogonal to |34𝒩⟩ and $a_{i}^{†}|A\rangle$. In turn, the overlap matrix $\left\langle \beta_{⊥}|\alpha_{⊥}\right\rangle$ is a full matrix, even though the overlap matrix ⟨β|α⟩ is diagonal for FE‐DVR. However, the full part of the matrix has rank I and its inverse can be applied efficiently using the Woodbury formula [23]. Even after orthogonalization against |i⟩ the expansion (2) remains over‐complete by construction. With finite sets of |A⟩ and |α⟩ this leads to near linear dependencies, but the space of near‐zeros remains small and suitable pseudo‐inverses can be applied at low cost, again employing the Woodbury formula, see discussion in [18].

### 2.2 Construction of Operators

Matrix elements between the three types of basis functions |34𝒩⟩, $\left|a_{i}^{†}A\right\rangle$, and $\left|a_{\alpha }^{†}A\right\rangle$ need to be evaluated for single‐particle and two‐particle operators

(5) $$S=S_{\mu ,\nu }a_{\mu }^{†}a_{\nu },\;T=T_{\mu \kappa ,\nu \lambda }a_{\mu }^{†}a_{\kappa }^{†}a_{\nu }a_{\lambda }$$

with summation implied over μ,ν,κ,λ running through both, the molecular orbitals i and the α‐basis. This follows standard procedures, where one benefits from the orthogonality $\langle i|\alpha_{⊥}\rangle =0$. One obtains expressions of a standard form that involve the single‐particle and two‐particle reaction density matrices for the ionic states

(6) $$\rho_{m,n}^{AB}=\langle A|a_{m}^{†}a_{n}|B\rangle ,\;\rho_{mk,nl}^{AB}=\langle A|a_{m}^{†}a_{k}^{†}a_{n}a_{l}|B\rangle$$

with only molecular indices m,n,k,l and generalized Dyson orbitals

(7) $$\eta_{m}^{\mathrm{34𝒩}A}=\left\langle \mathrm{34𝒩𝒩34𝒩𝒩𝒩}|a_{m}^{†}A\right\rangle ,\;\eta_{mk,n}^{\mathrm{34𝒩}A}=\left\langle \mathrm{34𝒩𝒩}|a_{m}^{†}a_{k}^{†}a_{n}A\right\rangle$$

However, as a general feature of the hybrid basis, higher reduced density and Dyson matrices are needed:

(8) $$\rho_{mku,nlv}^{AB}=\left\langle A|a_{m}^{†}a_{k}^{†}a_{u}^{†}a_{n}a_{l}a_{v}|B\right\rangle ,\;\eta_{mku,lv}^{\mathrm{34𝒩}A}=\left\langle \mathrm{34𝒩𝒩}|a_{m}^{†}a_{k}^{†}a_{u}^{†}a_{l}a_{v}A\right\rangle$$

These appear when evaluating two‐particle operators for the $\left|a_{i}^{†}A\right\rangle$ part of the basis and between neutral |3434𝒩⟩ and $\left|a_{i}^{†}A\right\rangle$ functions.

Such more general objects are not usually available directly from quantum chemistry codes. We were provided with a customized version of the COLUMBUS package [24], which outputs a full list of CI coefficients in terms of plain Slater determinants. From that, the above density and Dyson matrices are computed in a straight forward way following Slater‐Condon rules. The brute force approach is computationally intensive, but can be trivially parallelized by distributing the determinants. Still, depending on the exact CI variant selected in the COLUMBUS calculation, millions of determinants with non‐zero coefficient may appear and elimination of near‐zeros is needed to keep compute times under control. With the MCSCF‐MRCI states used for the present work, we obtained acceptable accuracies with CI basis sizes $≲10^{5}$ and truncation was not required. The generalized density and Dyson matrices are computed once for each set of states and saved for re‐use.

### 2.3 Calculation of Integrals

Another technical challenge of our hybrid approach is posed by integrals involving both, multi‐centered Gaussians and the numerical α‐basis. Rather than computing integrals with the primitive Gaussians and the α‐basis, we expand the molecular orbitals into a very large single‐centered γ‐basis, which is of the same type as the α‐basis and shares with it the finite‐element boundaries. The angular and radial orders of the γ‐basis are automatically pushed to convergence by monitoring kinetic energy matrix elements. Radial shells $\left[r_{n},r_{n+1}\right]$ that include nuclear positions are chosen to be narrow. In those shells, angular expansions of typically L≲60 appear, which far exceed the angular expansion in the α‐basis. Outside those shells angular momenta quickly drop off.

Having expressed the molecular orbitals in the γ‐basis, we can use the standard procedures of tRecX for evaluating all matrix elements for single‐ and two‐particle potentials, kinetic energy, and field interaction. Integrals involving only Gaussians are taken directly from COLUMBUS and are also used for crosschecking of the γ‐basis convergence.

The higher order density and Dyson matrices and the two particle operators $T_{\mu \kappa ,\nu \lambda }$, where the index ranges includes the α‐basis, cannot be kept in memory. Rather, two‐particle operator matrix elements of the haCC basis functions are directly integrated on a quadrature grid for the γ basis. This procedure is parallelized across the quadrature points, but still remains time‐consuming during initial setup. Results are saved for reuse with a hash for the exact definition of the operator and the expansion.

### 2.4 Computation of Photoelectron Spectra

When photoelectrons are ejected from the molecule by a finite pulse, depending on their energy, they can travel large distances while the pulse is still present. Given the large spatial extension of the pulse compared to the molecule, or, more technically, the infinite range of the dipole interaction, the electron energy is not fixed until the pulse is over. For a standard spectral analysis one would need to preserve the complete wave function until the end of the pulse. At longer pulse durations, wave functions can easily reach radii of 100s or 1000s of atomic units before the pulse is over. However, the influence of the molecular potential is quickly diminished beyond some 10s of atomic units and the electronic motion in the dipole‐field alone is known in closed analytic form. Using these observations, one can obtain the spectrum by properly accumulating over all times the electronic flux appearing at some surface outside the reach of the molecular potential. The wave function beyond the surface may be absorbed using a suitable absorber. This is the essence of the time‐dependent surface flux (tSurff) method [19, 20].

The key advantage of tSurff is that one can use a small simulation “box” with the radial grid dimensions of $R_{c}≲100$ au even when the actual wave function would expand to several orders of magnitude larger sizes. For short pulses, significant parts of the wave function may remain within that box. These will predominantly be low energy electrons, or, in case of resonances, electrons in slowly decaying resonance states, whose decay times far exceed the duration of the exciting pulse. For those cases, one can perform the time‐integration from the end of the pulse to infinity in analytic form at the expense of repeatedly solving a large linear system. This was observed in Reference [25] and given the name iSurf.

We define the spectral amplitude for photo‐emission into channel A with asymptotic electron momentum $\vec{k}$ as

(9) $$b_{A,\vec{k}}(t=\infty )=\langle \chi_{A,\vec{k}}|\Psi (t=\infty )\rangle$$

where $\chi_{A,\vec{k}}$ is the scattering solution for the full problem with outgoing momentum $\vec{k}$ in the A channel and δ‐normalization

(10) $$\left\langle \chi_{A,\vec{k}}|\chi_{A,{\vec{k}}^{\prime }}\rangle =\delta^{\left(3\right)}(\vec{k}-{\vec{k}}^{\prime }\right)$$

The tSurff spectral amplitude $b_{A,\vec{k}}(T)$ is given by the integral up to time T

(11) $$b_{A,\vec{k}}(T)=N\underset{B}{\sum }U_{AB}^{(\mathrm{ion})\;†}(T)\int_{0}^{T}dt\langle B,\vec{k},t|C(t)|\Psi (t)\rangle$$

with the antisymmetric product $\left\langle B,\vec{k},t|:=\langle B,t|\wedge \langle \vec{k},t\right|$ of an ionic factor with a single‐electron factor $\left\langle \vec{k},t\right|$. The time‐evolution of the ionic factor |A,t⟩ accounts for the dipole coupling between the ionic states

(12) $$\left|A,t\rangle =\underset{B}{\sum }U_{AB}^{(\mathrm{ion})\;†}(t)|B,0\right\rangle$$

and $\left|\vec{k},t\right\rangle$ is the Volkov solution for a free electron in the dipole field, with plane wave initial condition at time t=0 (in velocity gauge)

(13) $$\left|\vec{k},t\rangle =\exp\left\{-i\int_{0}^{t}\frac{1}{2}{\left[\vec{k}-\vec{A}(\tau )\right]}^{2}d\tau \right\}|\vec{k},0\right\rangle$$

Here $\vec{A}(\tau )=\int_{-\infty }^{\tau }\vec{\varepsilon }(\tau^{\prime })d\tau^{\prime }$ is proportional to the vector potential in Coulomb gauge for the dipole field $\vec{\varepsilon }$ and the solutions are δ‐normalized:

(14) $$\left\langle {\vec{k}}^{\prime },t|\vec{k},t\rangle =\delta^{\left(3\right)}(\vec{k}-{\vec{k}}^{\prime }\right)$$

The flux operator w.r.t. electron coordinate ${\vec{r}}_{N}$ is time‐dependent

(15) $$C(t)=\left[-\frac{1}{2}\nabla_{N}^{2}+i\vec{A}(t)\cdot {\vec{\nabla }}_{N},\Theta (|\vec{r}{|}_{N}-R_{c})\right]$$

where the commutator of the derivatives with the Heaviside function Θ restricts contributions to the surface $|{\vec{r}}_{N}|=R_{c}$. A derivation and further discussion of Equation (11) can be found in Reference [18] and references therein.

After the end of the pulse $\vec{A}(t>T)\equiv 0$, $H_{I}(t>T)\equiv 0$, the surface flux operator reduces to the standard time‐independent one and the time‐dependencies of the wave functions simplify to

(16) $$\begin{array}{ll} \left|A,\vec{k},t\right\rangle & =e^{-i(t-T)(E_{A}+{\vec{k}}^{2}/2)}|A,\vec{k},T\rangle \;\\ \left|\Psi (t)\right\rangle & =e^{-i(t-T)H_{0}}|\Psi ,T\rangle \; \end{array}$$

where $E_{A}$ is the energy of the ionic state. With that, the integration in Equation (11) can be extended in closed form to t=∞ resulting in [18, 25]

(18) $$b_{A,\vec{k}}(t=\infty )=b_{A,\vec{k}}(T)-i\langle A,\vec{k},T|C{\left(H_{0}-E_{A}-\frac{1}{2}k^{2}\right)}^{-1}|\Psi (T)\rangle$$

Note that the inverse is well‐defined as an absorber is used, which makes the Hamiltonian matrix $H_{0}$ non‐hermitian with all continuous spectrum strictly in the lower complex half‐plane.

#### 2.4.1 Absorption—Infinite Range Exterior Complex Scaling

As one discards $\Psi (\vec{r},t)$ outside the simulation box, one needs to carefully avoid any reflections or other distortions of the solution. To this end, we use infinite range exterior complex scaling [25], which is both mathematically well founded and computationally efficient. Exterior complex scaling is a method of analytically continuing the Hamiltonian H to a complex scaled $H_{\theta }$ by substituting the real coordinates in H with coordinates in the complex plane:

(19) $$\begin{array}{c} \vec{r}\to \left\{\begin{array}{cc} \vec{r} & \text{for}\;|\vec{r}|\leq R_{c}\\ \frac{\vec{r}}{\left|\vec{r}\right|}\left[R_{c}+e^{i\theta }(|\vec{r}|-R_{c})\right] & \text{for}\;|\vec{r}|>R_{c},\;0<\theta <\pi /2 \end{array}\right. \end{array}$$

If analyticity can be ensured, solutions $\Psi_{\theta }(\vec{r},t)$ obtained for $H_{\theta }$ agree with the physical solution $\Psi (\vec{r},t)$ for $|\vec{r}|\leq R_{c}$. Outside that region, the solution becomes exponentially damped, which makes it well representable on a square‐integrable basis. Complex scaling has the following remarkable properties of leaving bound state energies strictly unchanged and exposing resonance energies as discrete eigenvalues in the lower half complex energy plane, which, once exposed, remain independent of θ. This means, in particular, thresholds and resonance positions are not affected by complex scaling. Finally, continuous energies rotate into the lower half complex plane, which creates the desired absorption and leads to a decrease of overall norm under the non‐unitary time‐evolution $\exp(\;-itH_{\theta })$.

For numerical implementation we use a single special set of exponentially damped DVR functions on the last, infinite interval $\left[R_{c},\infty \right)$

(20) $$\begin{array}{c} \xi_{j}(r)=a_{j}(r)\exp(\;-\mu r),0<\alpha ,\;j=0,...,J\;\; \end{array}$$

where the $a_{j}$ are Lagrange polynomials at quadrature points on $\left[R_{c},\infty \right)$ that are suitable for the exponentially damped function. Note that complex scaling changes the *operator* $H_{\theta }$, which still acts on the standard $L^{2}(d^{3}r,{\mathbb{R}}^{3})$ Hilber space. Therefore also the argument of $\xi_{j}(r)$ remains real, $r\in [R_{c},\infty )$.

With small values of μ∼0.1 this basis can represent the long, exponentially damped tails of the high lying Rydberg states that appear in the Hopfield series of resonances. The exact choice for the value of θ is uncritical and we find our results to be invariant for θ=0.1 to 0.3.

#### 2.4.2 Coulomb Effects at Low Photo‐Electron Energies

The tSurff scheme as presented above is based on the Volkov solutions Equation (13), that is, the approximation that beyond the box size $R_{c}$ the potential does not affect the time‐evolution of the electron in the electric dipole field. With the long‐range Coulomb tail, this assumption is not strictly valid and creates numerical artifacts in the form of $R_{c}$‐dependent modulations of the photoelectron spectrum at energies $E≲1/R_{c}$.

A clean general solution to this problem would be to replace the Volkov solutions with time‐dependent solutions that include scattering in the asymptotic Coulomb potential. However, no analytic form is known for these solutions.

In the present case of very short pulses, the problem can be circumvented by observing that the pulse ends before any relevant flux reaches $R_{c}$. Once the pulse is over, we *do* know the time‐evolution: In a spherical expansion it is

(21) $$|k,l,m,t\rangle =e^{-itk^{2}/2}\frac{e^{i\sigma_{l}}F_{l}(1/k,kr)}{kr}$$

where m is the magnetic quantum number and $\sigma_{l}$ and $F_{l}$ are the Coulomb phase shift and the standard Coulomb wave function, respectively, for quantum number l. With these solutions replacing the Volkov solutions, there appear to be no artifacts at low energies.

### 2.5 Solving $\left(H_{0}-E)|\Phi \rangle =|\Psi (T)\right\rangle$

With dimensions up $≲2\times 10^{4}$ in the haCC basis, the linear system is not exceedingly large, but it needs to be solved repeatedly on a dense grid of energies $E=E_{A}+{\vec{k}}^{2}/2$ and solutions by direct methods become expensive. For iterative methods, a good preconditioner is needed, where the standard choices such as using a submatrix of $H_{0}$ with finite band width were found to be insufficient. This is likely related to the very non‐local nature of the expansion (2). Instead, we found a satisfactory compromise in using the full inverse ${\left(H_{0}-E_{0}\right)}^{-1}$ with $E_{0}$ near the region of interest as a preconditioner. One advantage of that preconditioner is that the Krylov space depends only on $E_{0}$, but not on E, as one readily sees from the identity

(22) $${\left(H_{0}-E_{0}\right)}^{-1}(H_{0}-E)=1+(E_{0}-E){\left(H_{0}-E_{0}\right)}^{-1}$$

Thus, with solutions of the full system for a small number of different $E_{0}$, spectra can be computed on a dense grid of energies at little additional cost [26].

In the calculations below, using a standard GMRES scheme, we found that a fixed Krylov subspace of dimension 40 would allow convergence over a range of ∼0.05 eV in the vicinity of a resonance peak and an overall number of ≲50 shift‐invert operations were needed for all 3000 spectral points in the energy interval from threshold at 0 up to 4 eV.

### 2.6 Single‐Photon Absorption Spectra

Single‐photon absorption as a function of photon energy is obtained from the photoelectron‐emission spectra of a broadband pulse by dividing the photoelectron spectra by the pulse's spectral intensity and summing over all channels.

The photoelectron spectral density $\sigma_{A}(E;\Omega )$ at photoelectron energy E in channel A is related to the spectral amplitude $b_{\vec{k}}(t=\infty )$ by

(23) $$\sigma_{A}(E;\Omega )=|\vec{k}|\int d\hat{k}|b_{A,\vec{k}}(t=\infty ){|}^{2}$$

where we integrate over emission directions $\hat{k}$. Here we have added the orientation of the molecular axis Ω=(Φ,Θ) w.r.t. the coordinate system of the laser field as an external parameter. For laser intensities where first‐order perturbation theory is applicable, one needs to calculate photoelectron spectra at 3 different orientations Ω, from which the complete Ω‐dependence can be reconstructed. In the case of cylindrically symmetric molecules and linear polarization, two such calculations are sufficient, for example, at parallel and perpendicular alignment of the axis relative to the laser coordinate system: denoting as Θ the angle between the laser polarization direction and the molecular axis, we see

(24) $$b_{A_{\Theta }\vec{k}}(t)=\cos(\Theta )b_{A_{0}\vec{k}}(t)+\sin(\Theta )b_{A_{\pi /2}\vec{k}}(t)$$

The idea can be applied to higher orders of perturbation theory with polynomial dependence on the external parameters: From a sufficient number of values at independent parameters, intermediate values can be computed by polynomial interpolation.

The absorption cross‐section $\sigma_{\text{abs}}(\omega )$ at photon frequency ω for a random mixture of orientations is obtained by summing over all channels and averaging over molecular orientations

(25) $$\sigma_{\text{abs}}(\omega )=\frac{\omega }{4\pi I(\omega )}\underset{A}{\sum }\int d\Omega \sigma_{A}(\omega -I_{A};\Omega )$$

where I(ω) is the spectral intensity of the pulse at photon energy ω, $I_{A}$ is the ionization potential for channel A, and the sum runs over all channels with $I_{A}<\omega$. The angular integrations can conveniently be performed by exact numerical quadratures in the variables (Φ,cosΘ).

## 3 Results

Photo‐absorption and photoelectron spectra for the $N_{2}$ molecule from the $X^{1}\Sigma_{g}^{+}$ ground state were calculated including the ground, first and second excited ionic channels—$X^{2}\Sigma_{g}^{+}$, $A^{2}\Pi_{u}$ and $B^{2}\Sigma_{u}^{+}$ channels, labeled as X,A, and B in the following. Both, ground and ionic excited states were calculated at the equilibrium internuclear distance of the neutral molecule (1.1 Angstroms). This is justified because during the short time of the ionization process, the nuclei can be considered as fixed. Relaxation into the ionic equilibrium and excitation of vibrational states is not included in our description. We expect the main effects through variation of the ionization potential. The possible impact of variations in ionization potentials is discussed below.

**FIGURE 1:**
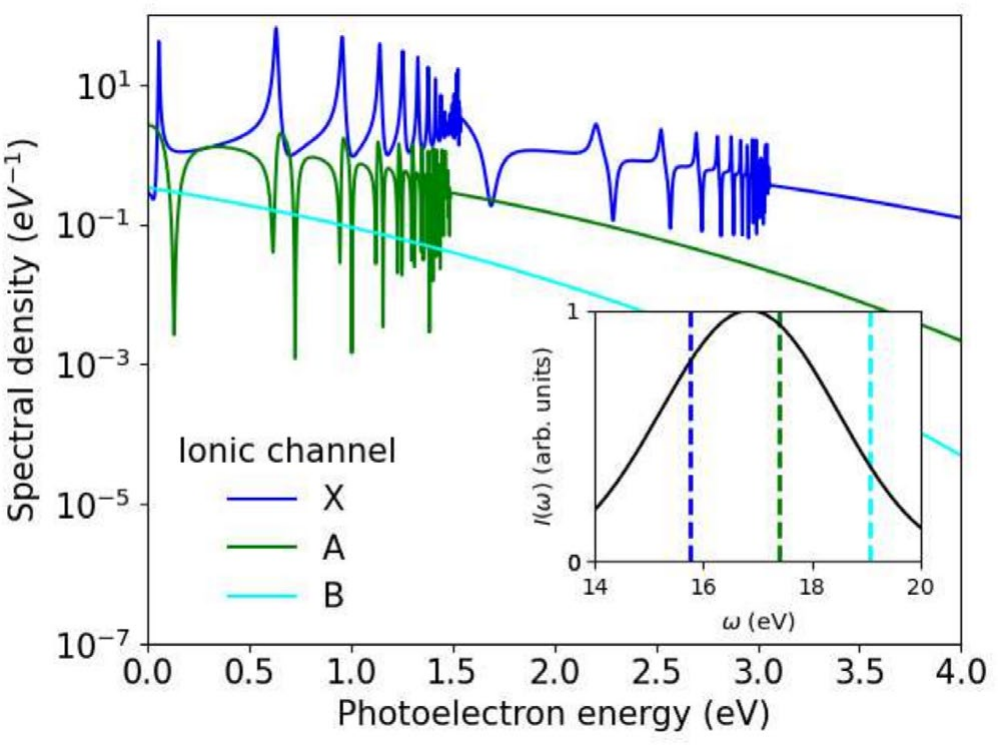
Single‐photon emission into the X, A, and B ionic channels with polarization parallel to the molecular axis. The inset shows the spectral intensity distribution of the pulse used in the calculation, with the channel thresholds indicated by dashed lines.

For the α‐basis Equation (3) we included angular momenta up to l,|m|≤9, which supports up to 9 photon transitions when starting from an m=0 state. The results presented in Figures 1 through 4 were computed with a radial box size of $R_{c}=100$ and MRCI states with the aug‐cc‐pvtz primitive basis described below. The exchange term of the bound molecular orbitals and the emitted electron is negligible beyond ∼30 au. The largest total basis size was ≈28,000. This includes J=50 complex scaling functions with exponential damping by $e^{-0.1r}$ starting at $R_{c}$ for each partial wave. The complex scaling angle was cosθ=0.1. Further details of the expansion and convergence are discussed in Section 3.3.

### 3.1 Single‐Photon Absorption in the Vicinity of the Hopfield Series

Our laser pulse parameters are central wavelength of 75 nm, FWHM duration of two optical cycles, and a pulse envelope shape of the form $\cos^{8}$, which produces a smooth, Gaussian‐like spectral intensity distribution. The peak intensity of $10^{13}\;{\text{W/cm}}^{2}$ is high in terms of experimental parameters but remains in the regime where perturbation theory is applicable (see Figure 6 in Section 3.3).

Figure 1 shows channel‐resolved photoemission at parallel alignment of the molecular axis with the linearly polarized laser field. The second series of resonances in the X‐channel and the series in the A‐channel both correspond to decay from the same set of doubly excited states and the same range of photon energies. The resonances are below the ionization threshold of the B‐channel. Note the pronounced differences in line shape between all three groups of resonances. Where the lines originate from the same resonances, the difference reflects the different phase and structure of the channel continuum.

**FIGURE 2:**
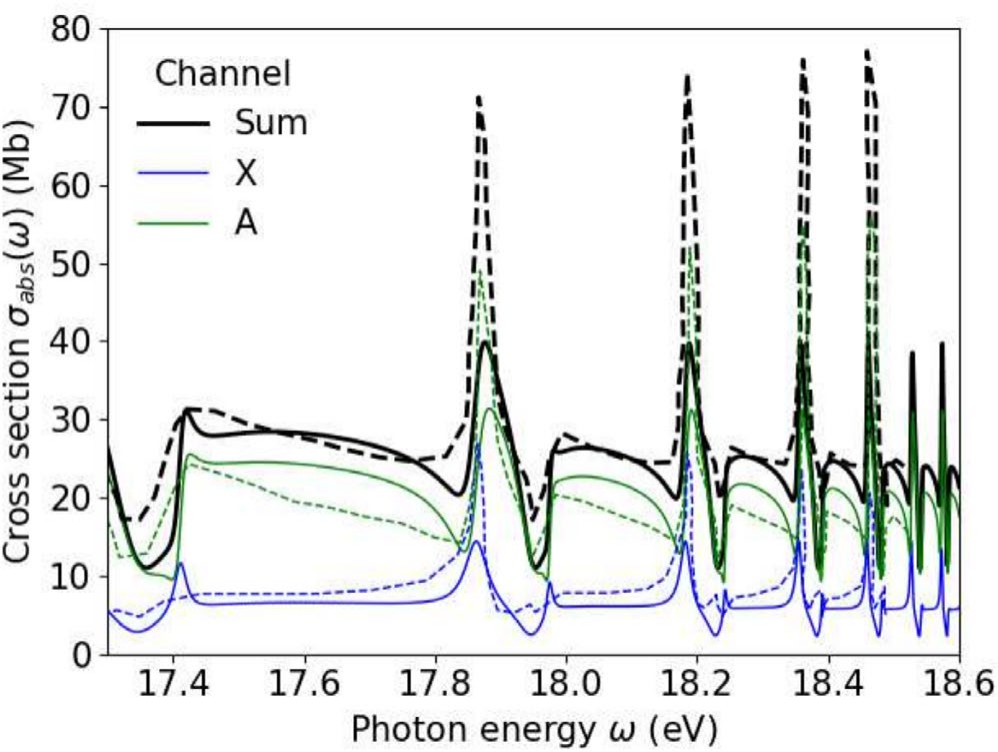
Photo‐absorption of $N_{2}$ with resonances of the Hopfield series. Our results (solid lines) and results from Reference [13], Figure 2b (dashed, data digitized from the figure).

For absorption spectra, we use the spectral amplitudes underlying Figure 1 and the corresponding amplitudes for perpendicular orientation, to obtain the cross‐section at arbitrary orientation Ω and to evaluate the orientation‐averaged cross‐section $\sigma_{\text{abs}}$ according to Equation (25). Results are given in Figure 2 together with cross sections from Reference [13]. Note that we have shifted the energy axis to 17 eV to match the experimental ionization threshold for the A‐channel, as in Reference [13]. The two calculations agree in magnitude, overall structure, general resonance shape, and resonance positions. However, there are minor differences: In our results, peaks are broader with a corresponding reduction in peak maximum in general, and between peaks there are more distinct features, in particular the dips to the right of the peaks are deeper. Total cross section between the resonance peaks agree well overall, but the cross sections are distributed differently between the X‐ and A‐channels.

Given that both methods use a similar general approach, the differences need some comment. Both channels, X and A, contribute to the dips to the left of the peaks. The broader resonances testify of stronger coupling to the continuum in our calculation. Thus, one possible source of the differences may be the representation of the continuum in the region of the CI states, which is by Gaussians in the case of Reference [13] versus in our case, molecular orbitals combined with purely numerical α‐basis. Another possible source of divergence is the CI states. We observe some dependence on the choice of the CI states used, but this only leads to changes in the absolute values of results on the scale of 25% and does not seem to affect the line shapes. For a more detailed discussion of these points, we are lacking here details on possible expansion dependencies in Reference [13] and therefore abstain from further speculation.

**FIGURE 3:**
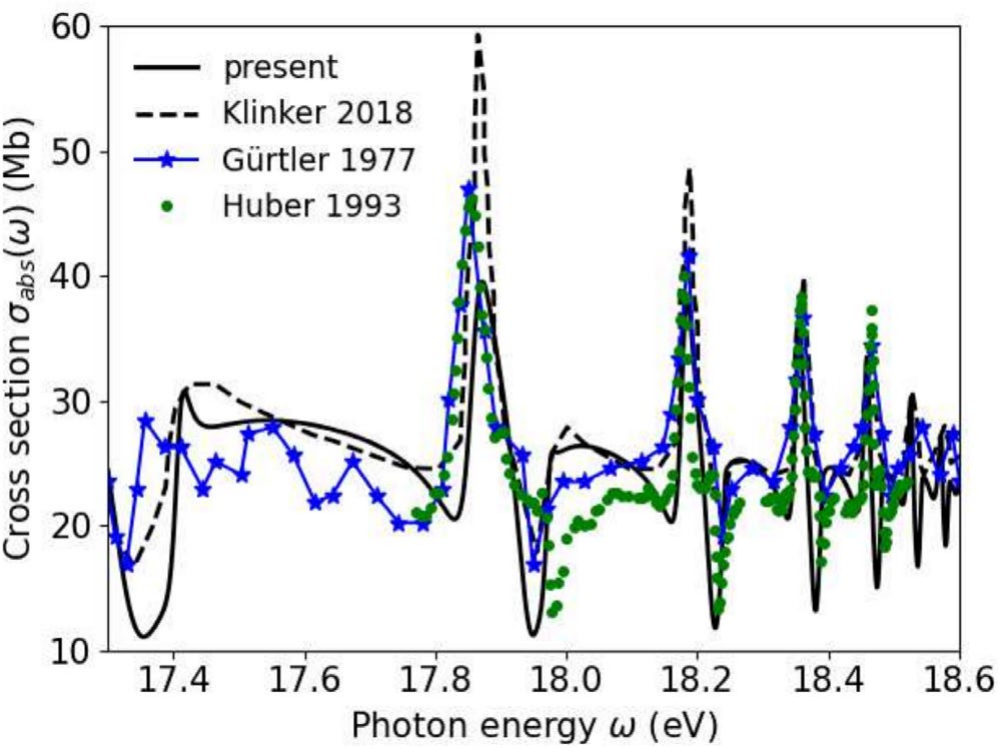
Photo‐absorption of $N_{2}$ near the Hopfield series. Our results (solid), synchrotron results from Gürtler et al. Reference [5] and Huber et al. Reference [6], and the calculation Reference [13] (dashed, data digitized from their Figure 3b). Both theoretical results are broadened by 0.015 eV to account for experimental resolution.

In Figure 3 we compare our results to synchrotron data taken from References [5, 6], where we account for the experimental resolution by convoluting the spectra of Figure 2 with a Gaussian of width 0.015 eV FWHM. Most importantly, the overall structure with peak heights decreasing with energy is reproduced. The dips to the right of the peaks are rather pronounced in our calculation and are also found in the experimental data.

### 3.2 Three‐Photon Excitation of Hopfield Resonances

**FIGURE 4:**
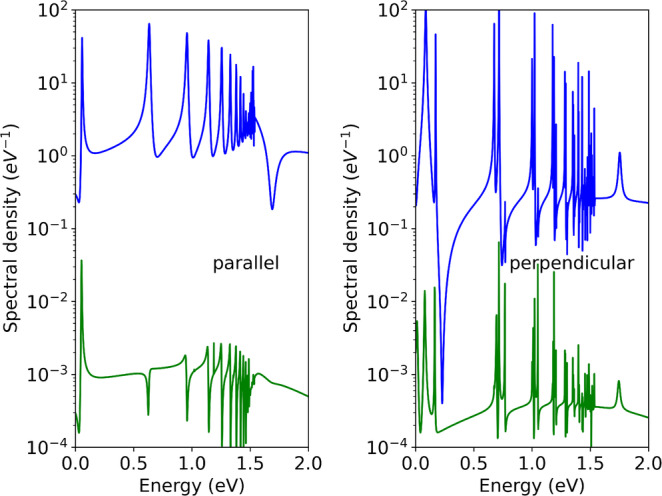
Dependence of photoemission line shapes on the photon number. Line shapes for with parallel alignment of the molecular axis (left) strongly differ between the single‐photon (upper curve) and the three‐photon process (lower curve). The difference is less pronounced for perpendicular alignment. The pronounced spike at 1.2 eV in the three‐photon curve for parallel alignment is due to an extremely narrow resonance.

Three‐photon transitions at the tripled wavelength of 225 nm access the same series of resonances as single‐photon transitions at 75 nm. Figure 4 compares the photoelectron emission spectrum $\sigma_{\text{tot}}=\sum_{A}\sigma_{A}$ with $\sigma_{A}$ of Equation (23) for the two wave lengths. We use the same pulse shape and two‐cycle duration with the same perturbative intensity of $10^{13}\;{\text{W/cm}}^{2}$ for both.

Being in the perturbative regime, the three‐photon transition is strongly suppressed compared to the single‐photon transition by more than three orders of magnitude. As to be expected, the main resonances all appear in both spectra, but the line shapes change drastically. The effect is the biggest for parallel alignment and less pronounced for perpendicular alignment, Figure 4.

On general terms, such changes are to be expected, as Fano line shapes are determined by the final continuum state, in particular on its phase relative to the resonant state. With its ionization pathway through—in the language of perturbation theory—two levels of virtual intermediate states, both, relative phase and the angular distribution of the continuum state will differ. A more detailed study of that pathway and the reason for the apparently stronger effect in parallel alignment are outside the scope of the present work.

### 3.3 Basis Dependence and Convergence

We discuss, for wavelengths 75 and 225 nm, the dependence of our results on the most critical discretization choices: The CI states, the simulation box size, and the angular size of the α‐basis.

The CI states were calculated using COLUMBUS [24]. We obtain molecular orbitals in MCSCF with averaging performed over the neutral and X, A, and B ionic states. CI states were built using the MRCI routine of COLUMBUS. Using the Gaussians augmented, correlation consistent bases, we see a significant variation of energies when going from the triple (aug‐cc‐pvtz) to the quadruple (aug‐cc‐pvqz) zeta‐basis. Table 1 shows the ionization energies obtained with both sets and experimental values.

**TABLE 1:** Ionization potentials (in eV) for the X, A, and B channels for the two primitive bases aug‐cc‐pvtz (triple) and aug‐cc‐pvqz (quadruple), comparing to measured values from Reference [27].

Basis	X	A	B
Triple	16.03	17.69	19.36
Quadruple	15.75	17.4	19.08
Measured [27]	15.58	17.0	18.8

**FIGURE 5:**
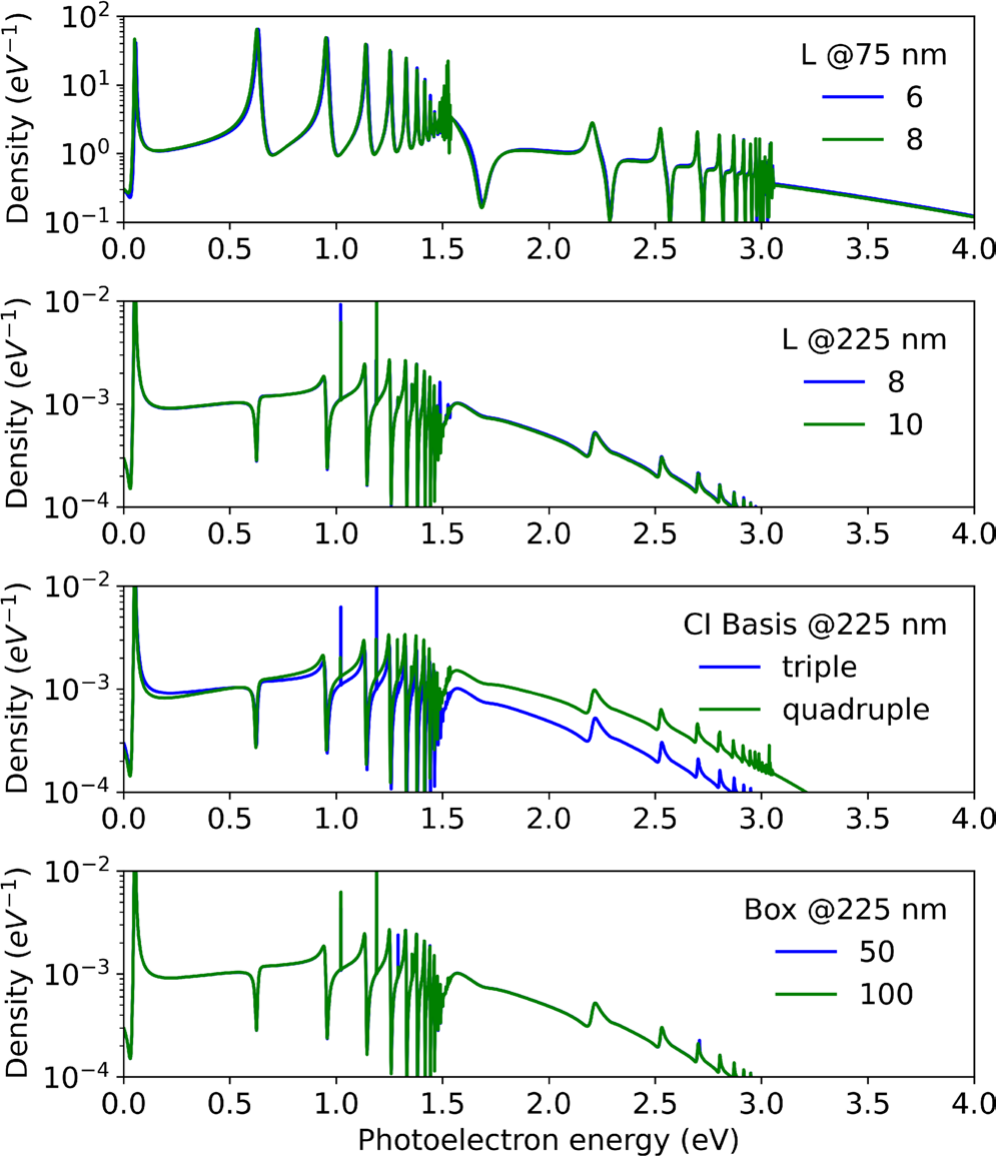
Dependence on the CI basis, angular expansion L, and box size $R_{c}$. Density in the X‐channel with parallel alignment of the molecular axis with laser polarization. Wavelengths 75 and 225 nm, as indicated in the panels.

The maximal angular momentum $L\geq l_{\alpha }$ in the α‐basis was varied from L=6 to 10. Figure 5 shows the dependence of the spectra on L and the primitive basis. Sensitivity to L is low and does in no way affect any of the observations made above. Sensitivity to the primitive basis is somewhat larger. Single‐photon emission increases by about 20% when approaching the experimental values by moving the triple to the quadruple basis (not shown). The effect is more pronounced for the 225 nm wavelength, where at larger energy the difference in absolute value amounts to nearly a factor two. However, line shapes are barely affected by the shift.

At this point, we have no conclusive evidence whether changes in thresholds or in the wave function (or both) are responsible for the sensitivity to the primitive basis. In an attempt to adjust ionization potentials used in the time‐propagation to their experimental values, we have met massive artifacts, likely due to undetected technical reasons.

Our results are barely affected by the radius $R_{c}$ of the simulation box, as demonstrated in the lowest panel of the figure. The spectrum remains unchanged when reducing the box size from 100 au to 50. The only conspicuous difference in the graphs appears in the height of extremely narrow peaks, which however, is an aliasing effect due to the finite resolution of the energy grid combined with miniscule shifts in the narrow peaks.

**FIGURE 6:**
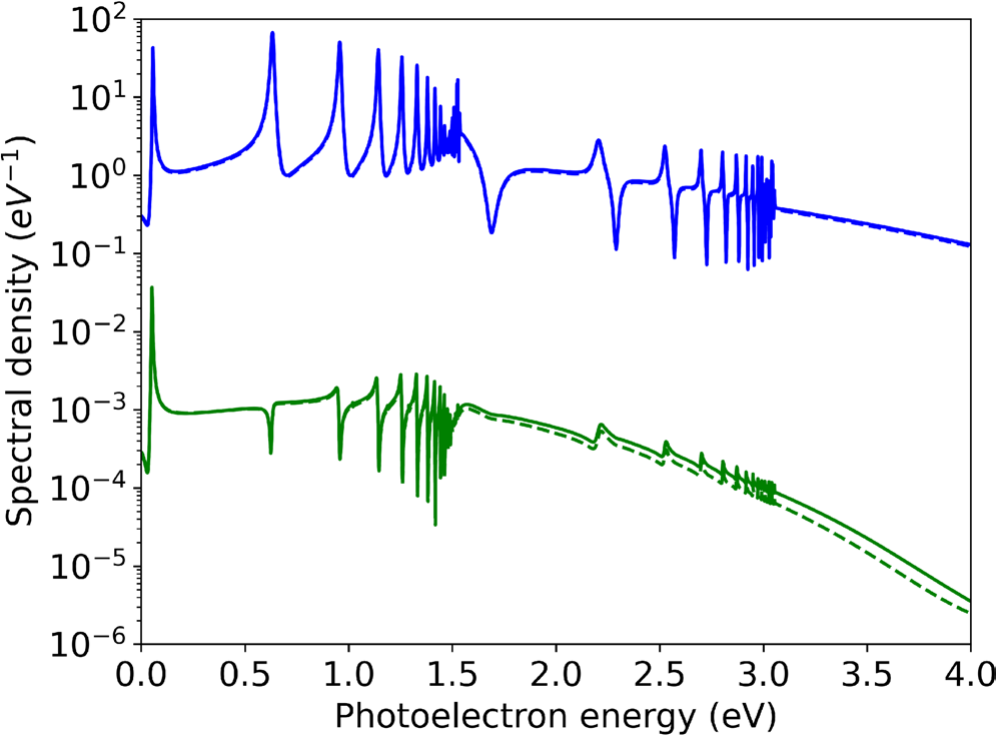
Perturbative scaling of the single‐photon (75 nm, blue lines) and three‐photon (225 nm, green lines) electron emission. The solid lines are for intensity $10^{13}\;{\text{W/cm}}^{2}$, the dashed lines for $10^{12}\;{\text{W/cm}}^{2}$ are scaled by 10 and $10^{3}$ for the 1‐ and 3‐photon cases, respectively. The curves coincide within the resolution of the graph for the single‐photon case, for three photons the onset of the 4‐photon transition leads to deviations above ≳1.5 eV.

Finally, in Figure 6 we demonstrate that the intensity is perturbative and emission densities scale as I and $I^{3}$ in the single‐ and three‐photon cases, respectively. For the three‐photon case, the results deviate from the expectations due to some contribution from 4‐photon transitions.

## 4 Conclusions

A recent extension of tRecX‐haCC to include the iSurf technique for computing photo‐emission from long‐lived resonances was introduced. Photo‐emission cross sections at fixed molecular orientation are extracted from a single solution of the time‐dependent Schrödinger equation using a broad band pulse. In the perturbative regime, spectra for arbitrary molecular alignment with respect to laser polarization can be obtained from a discrete number of different alignments, where the exact number depends on the perturbation order and the system's symmetry. In the case of the cylindrically symmetric molecule $N_{2}$, first‐order transitions, and linear polarization, two calculations at parallel and perpendicular alignment suffice. The scheme can readily be extended to n‐photon transitions in n'th order perturbation theory, where a correspondingly larger set of calculations is needed.

The extraction of photoelectron spectra involves solving a linear equation with the field free Hamiltonian shifted for each photoelectron energy. This is done iteratively, where the use of the full operator for preconditioning proved to be the key technical advancement in obtaining fine‐grained cross‐section data at comparatively low computational cost. We also discussed the techniques for evaluating the higher density and Dyson matrices appearing in the operators and for integrating the matrix elements involving both, the molecular and numerical basis, by which the operator setup times are kept in a manageable time.

Using these tools, we computed photoabsorption in the photon energy range including resonances of Hopfield series of $N_{2}$, for which precise synchrotron measurements and recent calculations are available. We found very satisfactory agreement of our results, with theoretical as well as experimental cross sections. Looking into details, our calculations reproduce some of the features seen in experiments that seem to have escaped the earlier calculation.

We then proceed to calculating three‐photon ionization around the same series of resonances. Here the time‐dependent approach plays out its advantage, as a third‐order perturbative calculation is disproportionately demanding, while there is no relevant increase in complexity for the time‐dependent approach. As the main observation, we find a pronounced dependence of the line shapes on the photon order. Similar results had been reported earlier [9], but a deeper theoretical analysis must be deferred to future work.

## Data Availability

The data that support the findings of this study are openly available in https://gitlab.physik.uni‐muenchen.de/AG‐Scrinzi/tRecX.git at https://gitlab.physik.uni‐muenchen.de/AG‐Scrinzi/tRecX.git.
